# Supplementary figures and images for: Succession of the intestinal bacterial community in Pacific bluefin tuna (Thunnus orientalis) larvae
Source: PLoS One. 2022 Oct 6;17(10):e0275211. doi: 10.1371/journal.pone.0275211 (PMC9536584; doi:10.1371/journal.pone.0275211)

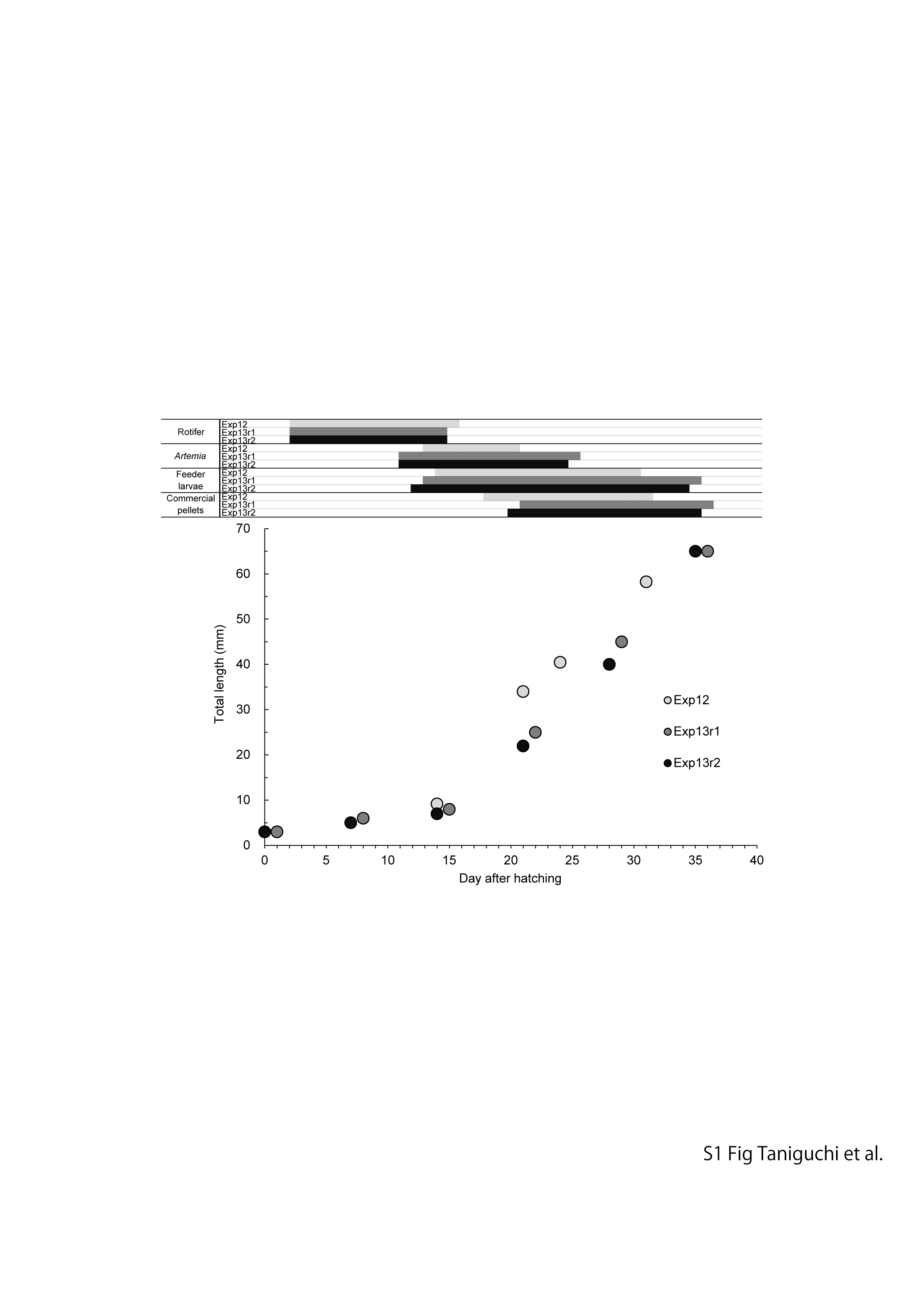

Supplement: S1 Fig — The growth of bluefin tuna is indicated as a plot graph, and the feeding schedules are shown as a bar graph at the top of the plot graph. Exp12, Exp13r1, and Exp13r2 indicate the year (2012 or 2013) and round (r1 or r2) in which the experiment was performed. (TIF) [file pone.0275211.s001.tif]

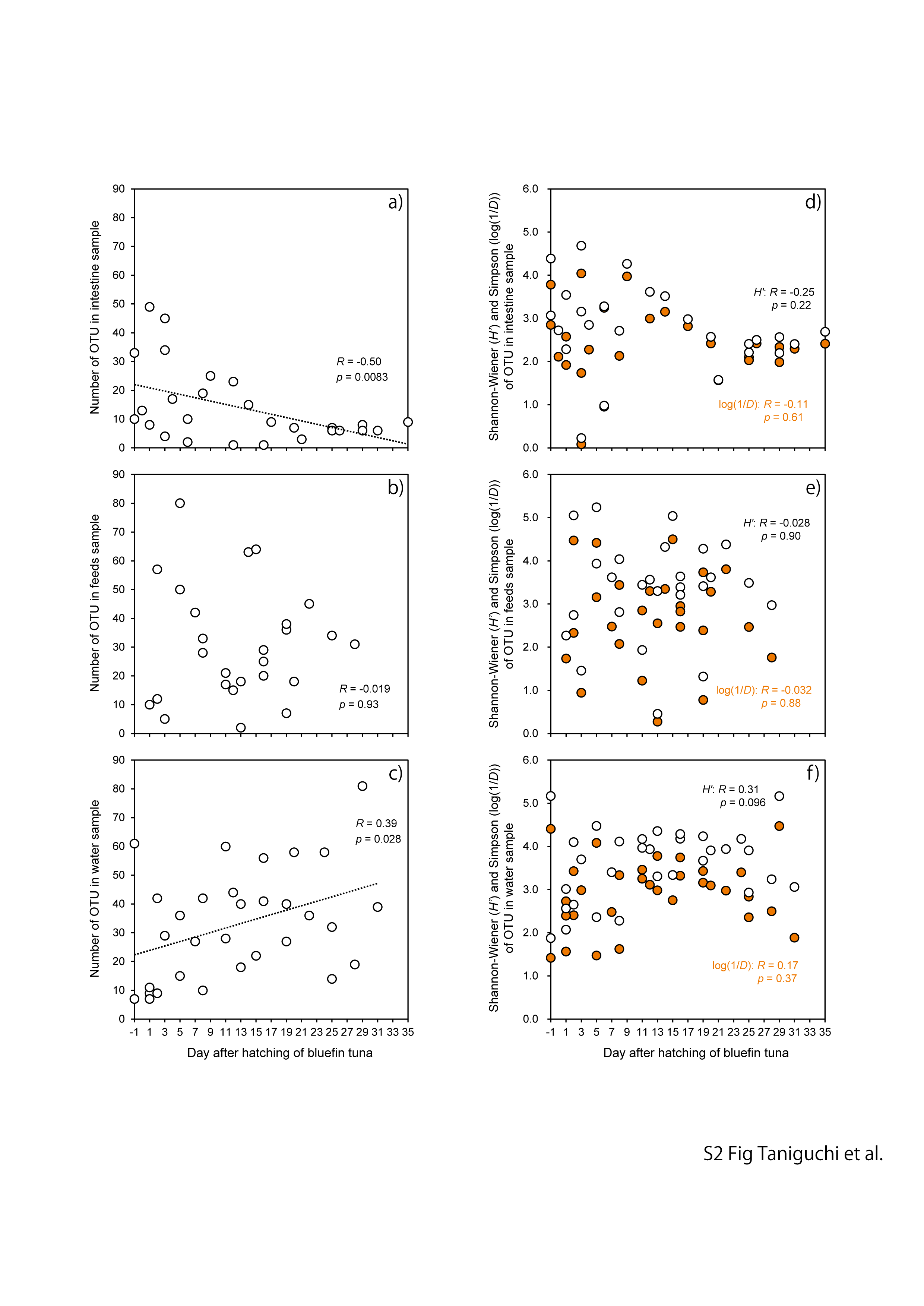

Supplement: S2 Fig — a)–c) Show the number of OTUs in intestine, feed, and water samples, respectively. d)–f) Show indices of the Shannon-Wiener (H’) (open circle) and Simpson (log(1/D)) (filled orange circle) in intestine, feed, and water samples, respectively. r and p values describe Pearson’s correlation. (TIF) [file pone.0275211.s002.tif]

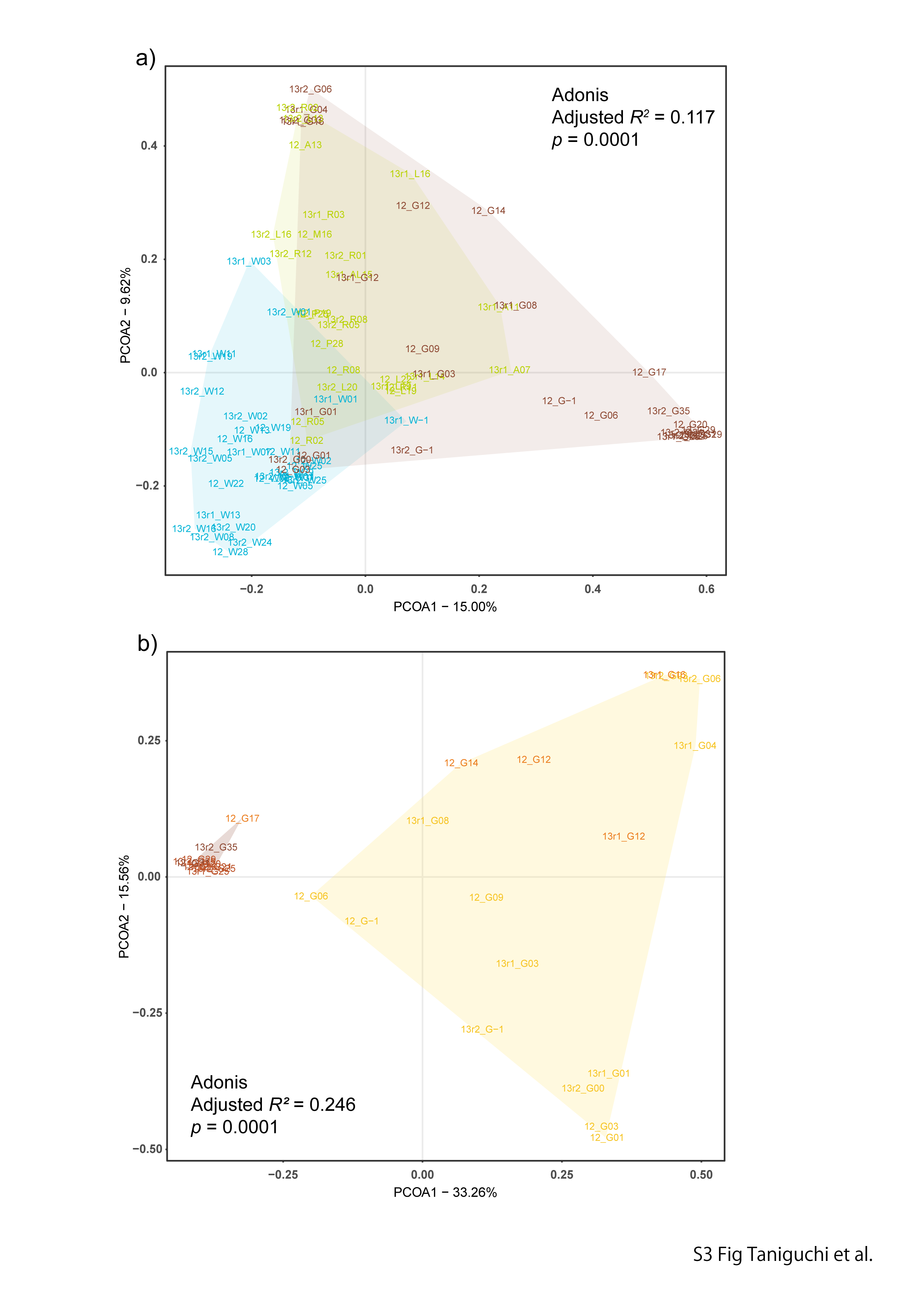

Supplement: S3 Fig — (a) PCoA constructed using all samples, rearing water (blue letters), feed (green letters) and intestine (brown letters). (b) PCoA constructed using only intestinal samples, the early (yellow group) and late (brown group) stages based on Adonis test (p = 0.0001). Sample names are represented by experimental year, sample type, and DAH of bluefin tuna; 0 DAH is the day of hatching and Day -1 represents the unhatched egg. For example, 12_G17 represents intestinal samples of 17 DAH in 2012. W, water; G, intestine; R, rotifer, A, Artemia; L, feeder larvae (striped beakfish); P, commercial pellet. (TIF) [file pone.0275211.s003.tif]

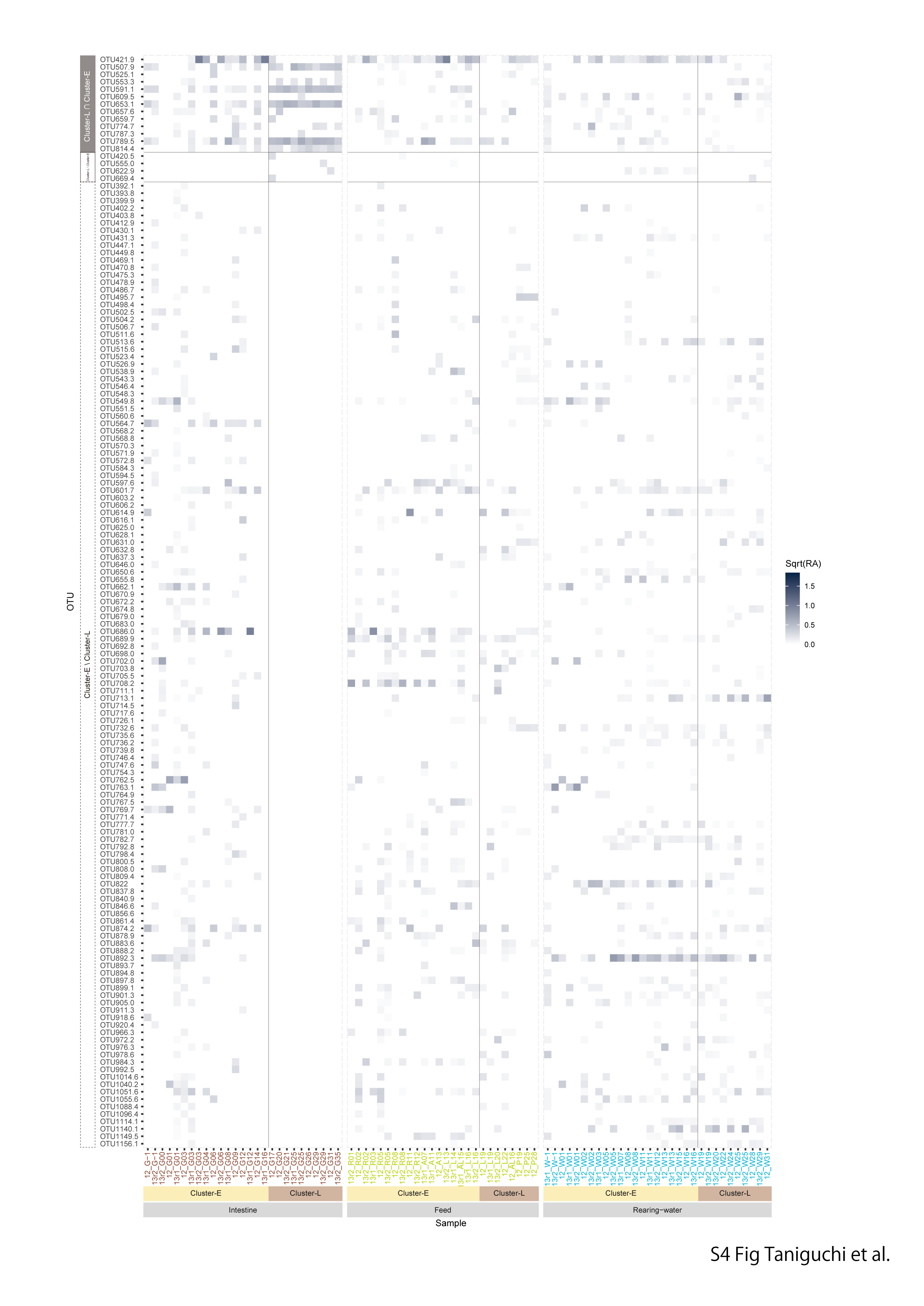

Supplement: S4 Fig — The heatmap color corresponds to the square root of transformed relative abundance of each OTU in each sample. Sample names are represented by experimental year, sample type (rearing water, blue letters; feeds, green letters; intestine, brown letters), and DAH of bluefin tuna; 0 DAH is the day of hatching and Day -1 represents the unhatched egg. For example, 12_G17 represents intestinal samples of 17 DAH in 2012. The order of sample is based on the hatching day of larvae. W, water; G, intestine; R, rotifer, A, Artemia; L, feeder larvae (striped beakfish); P, commercial pellet. (TIF) [file pone.0275211.s004.tif]
