# Supplementary material for: Succession of the intestinal bacterial community in Pacific bluefin tuna (Thunnus orientalis) larvae
Source: PLoS One. 2022 Oct 6;17(10):e0275211. doi: 10.1371/journal.pone.0275211 (PMC9536584; doi:10.1371/journal.pone.0275211)
Supplement: Attachment — Submitted filename: Response to Reiewers.docx [file pone.0275211.s007.docx]

PONE-D-21-35860

Succession of the intestinal bacterial community in Pacific bluefin tuna (*Thunnus orientalis*) larvae

PLOS ONE

1. The data cannot support the conclusions. PLOS ONE is designed to communicate primary scientific research, and welcome submissions in any applied discipline that will contribute to the base of scientific knowledge. But the data of this manuscript cannot support the conclusions.

> This paper presents a molecular ecological approach to the succession pattern of intestinal bacteria. We did not perform species identification because the main purpose of the present manuscript is to understand how environmental factors such as feed and rearing water influence intestinal microbiota. We hope this meets the aim of your journal.

2. This manuscript has the statistical analysis problem.

> We have addressed this problem.

3. The revised manuscript needs to address each of the comments of the reviewers.

> We have responded to the reviewers’ comments individually.

Reviewers' comments:

Reviewer's Responses to Questions

Comments to the Author

1. Is the manuscript technically sound, and do the data support the conclusions?

The manuscript must describe a technically sound piece of scientific research with data that supports the conclusions. Experiments must have been conducted rigorously, with appropriate controls, replication, and sample sizes. The conclusions must be drawn appropriately based on the data presented.

Reviewer #1: Partly

Reviewer #2: Partly

2. Has the statistical analysis been performed appropriately and rigorously?

Reviewer #1: N/A

Reviewer #2: I Don't Know

3. Have the authors made all data underlying the findings in their manuscript fully available?

The PLOS Data policy requires authors to make all data underlying the findings described in their manuscript fully available without restriction, with rare exception (please refer to the Data Availability Statement in the manuscript PDF file). The data should be provided as part of the manuscript or its supporting information, or deposited to a public repository. For example, in addition to summary statistics, the data points behind means, medians and variance measures should be available. If there are restrictions on publicly sharing data—e.g. participant privacy or use of data from a third party—those must be specified.

Reviewer #1: Yes

Reviewer #2: Yes

4. Is the manuscript presented in an intelligible fashion and written in standard English?

PLOS ONE does not copyedit accepted manuscripts, so the language in submitted articles must be clear, correct, and unambiguous. Any typographical or grammatical errors should be corrected at revision, so please note any specific errors here.

Reviewer #1: Yes

Reviewer #2: Yes

5. Review Comments to the Author

Please use the space provided to explain your answers to the questions above. You may also include additional comments for the author, including concerns about dual publication, research ethics, or publication ethics. (Please upload your review as an attachment if it exceeds 20,000 characters)

Reviewer #1: The author examined the gut microbiota of blue fin tuna from the offshore cage rearing system by Automated approach for ribosomal intergenic spacer (ARISA) analysis. However, even with dissimilarity tree analysis, this method can give only preliminary dominant microbiota change but not enough to state the solid succession limited by the lack of bacteria annotation and the multi-analysis of the microbiota change of feeds and rearing water due to seemingly technical issue of DNA extraction and the uneven distribution of bacteria abundance.

Major issue:

1. The biggest issue with this work is still the accuracy of ARISA. Have author(s) done any single 16s for getting idea on whether How different OTU number or major OTU number difference? Even though the author(s) claimed ARISA can represent major dominant bacteria?

> In this manuscript, we aimed to investigate how intestinal microbiota communities are formed with respect to microbes found in food and water. Therefore, we used ARISA, which is reported to be able to estimate microbial diversity and community composition in terms of OTUs (Fisher and Triplett, 1999). It is also a semi-quantitative technique (Brown et al, 2005) and has been utilized in many studies. In humans, it has been reported that the predominant bacteria have a significant impact on the host (Gerritsen et al, 2011), so understanding the microbial dynamics detected by ARISA will be very meaningful. However, as you pointed out, because species composition needs to be elucidated to utilize the data, a deep sequencing approach should be used in the future.

2. Figure 1. Can author(s) use PCoA to represent the beta-diversity of dominant bacteria from ARISA? PCoA can represent better than tree in terms of distinguishing the different microbiota group along with statistical analysis such as ANOSIM/ADONIS.

> Thank you for your suggestions. We performed PCoA and ADONIS (9999 permutations). These results are shown in supplementary Fig S3 because the eigenvalues of axes 1 & 2 were low, especially in PCoA of all samples (S3a Fig). The ADONIS results are also shown in Fig S3, which shows the adjusted *R^2^* of 0.117 with a *p* value of 0.0001 among sample types, and an adjusted *R^2^* of 0.213 with a *p* value of 0.0001 between Clusters E and L, indicating significant differences. Additionally, for the significance in the cluster analysis (Fig. 1), we performed SIMPROF analysis (9999 permutations with a p < 0.0001 according to ADONIS result). The results are shown in Fig. 1 as bars below each cluster. These results were similar to those yielded by PCoA and ADONIS.

3. Venn diagram: how were the 117 and 4 distinguished bacteria distributed in the cluster E and L samples respectively?

> Thank you for pointing this out. First, after rechecking all OTUs, we re-drew the Venn diagram (Fig. 2) because the OTU number detected from Cluster-E was wrong in the previous manuscript; the correct number was 130 OTUs, not 117 OTUs. The distribution of the 130 and 4 bacterial OTUs is shown in Fig S4.

4. It seems the type of feeding organisms changed during the seedling (Table. S1) and their microbiota could change (Fig. S2e). Can authors show how different the microbiota is between different feeding organisms? This will tell us how if the feeds can really impact the intestinal microbiota.

> Thank you for your comment. We showed the distribution in S4 Fig. Bacteria in feed sample are highly variable, but similar for the same feed items. Interestingly, especially during the feeding period of rotifers and Artemia, bacteria from them were detected as intestinal bacteria, but most of the bacteria were no longer detected after the period.

5. Figure 3. It seems a lot of OTUs (from 789.5- 774.7) were missing in the most of the rearing water and feed samples but presenting in either egg or larvae intestine. If so, then host factor can be more critical than feed/rearing water in microbiota succession?

> As you mentioned, we speculate that host factors, such as digestive enzymatic activity, are among the critical factors for determining microbiota composition. In this manuscript, we aimed to identify the general process of intestinal microbiota development, that is, to determine whether the microbiota come from water, feed items, or another source.

6. Line179-180. however, no PCR products were obtained from some intestinal samples. Could it be the issue of bacteria DNA extraction? if so, then this will also affect the diversity and total abundance of the microbiota.

> Unfortunately, DNA extraction did not work for some samples. However, the method in this manuscript (physical disruption by bead beating; chemical lysis by lysozyme, proteinase K and SDS; and phenol/chloroform purification) has been used for DNA extraction in many previous papers. Every step commonly reported for effective DNA extraction from intestinal samples (e.g., Daly et al., 2012; Yuan et al., 2012; Eun et al., 2014; Dubin et al., 2016; Han et al., 2018). One of the reasons why no PCR products were obtained from some intestinal samples could be low abundance of intestinal microbes during development of host digestive tract. In any case, as you mentioned, care should be taken in interpreting the results because DNA extraction could introduce bias with regard to microbial diversity.

Minor issue:

1. Figure S2. – Y-axis. Number of OTU instead of Number if OTU. P value of R2?

> Thank you for pointing this out. We have corrected “if” to “of” and showed *P-* values in S2 Fig.

Reviewer #2: The manuscript entitled “Succession of the intestinal bacterial community in Pacific bluefin tuna (Thunnus orientalis) larvae” is try to investigated the succession process of intestinal bacteria during larvae rearing and their relationship between rearing water and feed (rotifer, artemia, feeder larvae and commercial pellets. The bacterial community was detected by an “automated ribosomal intergenic spacer analysis (ARISA)” methods and the operational taxonomic unit (OTUs), logarithm of inverse Simpson and Shannon-Weiner indices were used as the diversity indices. I have to say that this experiment is difficult to perform due to the sample collection was difficult. This manuscript also may give us very different results. However, there are some issues still need to be clarified before this draft can be published.

> Thank you for your comments.

1. Since the fish samples were collected individually, how to define the data collected from these fish is succession?

> The fish were reared under proper management, such as water quality, feed quality and quantity, and the number of reared fish from hatching to net-cage transfer. Fish were maintained under the same physiological conditions in each trial. In this manuscript, the formation of similar intestinal microbiota in the three rounds was considered evidence of this. We also collected multiple fish for each sample to decrease the effect of individual differences. Therefore, although fish samples were collected individually, we feel that our data are sufficient to indicate succession.

2. I’m curious what is the OTUs number variation between each pooled sample?

> “Bacterial abundance in rearing water, feed, and intestine samples ranged from 7–81, 2 –80, and 1– 49 OTUs, respectively (Tables 1–3)” lines 195–196 in “Revised Manuscript with Tracked Change”. This variation likely occurred because the predominant bacteria changed during the development of the host gastrointestinal tract. In this manuscript, we standardized the DNA amount loaded (100 ng) into the ARISA instrument to reduce the variation between samples (line 168).

3. In the manuscript, the authors mentioned the bacterial community in fish larvae have great different in the late larvae stage (after 17 dph). Even the number of OTUs seems reduce after this stage. But how to define this observation.

> This probably occurs due to the development of the host gastrointestinal tract, particularly the increased activities of digestive enzymes. Only bacteria that can tolerate the intestinal environment can survive.

4. As I understand, the next generation sequencing (NGS) methods had also can using in analyse bacterial community composition in gut or environment for some years. Not only give you the different OTUs reactions, but also can give you their composition. How you compare the ARISA and NGS methods using in bacterial community ananlysis in your research.

> As you mentioned, the NGS method is one of the strongest approaches for analysis of intestinal microbiota. However, in this manuscript, we focused on the relationship between the intestinal bacteria and the bacteria found in the feed and rearing water, which does not necessarily require identification of bacterial species composition. In fact, using ARISA, which is more economical than NGS, it is easy to compare multiple samples. We identified the importance of egg management for the formation of intestinal microbiota in the late stage. When compared to NGS data, the trend in major species may not change because ARISA detects predominant bacteria. However, because NGS can detect rare species, it may provide different findings in this respect.

6. PLOS authors have the option to publish the peer review history of their article (what does this mean?). If published, this will include your full peer review and any attached files.

If you choose “no”, your identity will remain anonymous but your review may still be made public.

Do you want your identity to be public for this peer review? For information about this choice, including consent withdrawal, please see our Privacy Policy.

Reviewer #1: No

Reviewer #2: No
