# Supplementary material for: Succession of the intestinal bacterial community in Pacific bluefin tuna (Thunnus orientalis) larvae
Source: PLoS One. 2022 Oct 6;17(10):e0275211. doi: 10.1371/journal.pone.0275211 (PMC9536584; doi:10.1371/journal.pone.0275211)
Supplement: Attachment — Submitted filename: Response to Reiewers.docx [file pone.0275211.s008.docx]

PONE-D-21-35860R1

Succession of the intestinal bacterial community in Pacific bluefin tuna (*Thunnus orientalis*) larvae

PLOS ONE

1. The data cannot support the conclusions. PLOS ONE is designed to communicate primary scientific research, and welcome submissions in any applied discipline that will contribute to the base of scientific knowledge. But the data of this manuscript cannot support the conclusions. Some technical issues have to be resolved in the manuscript.

In this manuscript, we aimed to know how the pattern of intestinal bacteria change as the host fish grow. Hence, we think that the method ARISA and obtained data are enough to address it and support the conclusions.

2. This manuscript has the statistical analysis problem.

We have already addressed statistical problems pointed out by two reviewers. Reviewer #1, who initially indicated that there was a problem, no longer indicate it, and Reviewer's Responses to Questions #3 in this time is also “no problem”.

3. The revised manuscript needs to address each of the comments of the reviewers.

We have responded to the reviewers’ comments individually.

Reviewer #1: The revision has improved and given more clues in the potential bacterial community change. However, come technical issues have to be resolved or addressed in the context.

We would like to thank the Reviewer for the careful review of this manuscript.

1. ARISA: I thank the author for his/her detailed reply on how ARISA was used in previous studies. Nonetheless, most of the studies using ARISA were back in the time NGS was not widely used. The current bacterial studies would need NGS (16S rDNA) analysis to really point out the bacterial community change. It would be more technical sound if the author can show a data to indicate that each ARISA peak really represents one bacterial species/genera in order to reach NGS resolution. (Is the binning method good enough to separate single bacteria type?)

In this manuscript, we aimed to investigate how intestinal microbiota communities are formed with respect to microbes found in food and water. We know your point, however ARISA allows us for the rapid comparison with high-throughput and cost-effective, and can also yield meaningful and valuable results. Previous studies show that each ARISA peak represents one bacterial species or strain and the binning method good enough to separate single species (e.g., Brown et al. 2005, Kovacs et al. 2010). Also, several reports show ecological coherence of bacterial diversity pattern between ARISA and NGS techniques (e.g., Bienhold et al. 2011, Gobet et al. 2014, Jami et al. 2014).

Thus, as you suggested, we added one sentence on the ecological coherence between the techniques: lines 253–255 in Revised_Manuscript “It is also reported that there is ecological coherence of bacterial diversity pattern between ARISA and deep sequencing techniques [36–38]”.

2. Can the author explain how this is happening in my previous comment#4 regarding the highly variable bacterial community in feeds but later disappeared in the intestine?

We think this probably occurs due to the development of the host gastrointestinal tract, particularly the increased activities of digestive enzymes. Only bacteria that can tolerate the intestinal environment can survive and so the most of highly variable bacterial community in feeds cannot survive and colonize in the intestine (lines 281–294 in Revised_Manuscript).

3. Have author compared the ARISA peaks to parental fish from the early- and late-stage larva? I'm asking because part of the bacterial community may be acquired from the parents.

No, we haven’t. The specific bacteria in the late stage larvae, 17 days after hatching, were also found on egg surface, egg shell. The egg shells were floating in the rearing water for long time after hatching. If the vertical transmission of bacteria from the parent fish occurred, the bacteria from the parents might give an influence on the early stage of larvae. Also, to our knowledge, a bacterial vertical transmission has not yet been reported as for bluefin tuna and there is only one paper on betanodaviruses in bluefin tuna (Sugaya et al., 2009). Anyway, as we have no data about the intestinal bacteria of adult bluefin tuna, this point, the vertical transmission from the parents, should be discussed in the future study. Thank you.

4. Can the author discuss the error by DNA extraction and the resulting potential error in bacterial community diversity/richness regarding to my previous comment# 6?

This was the challenge in the past as we commented previously (e.g., Daly et al., 2012; Yuan et al., 2012; Eun et al., 2014; Dubin et al., 2016; Han et al., 2018). The results of DNA extraction are controversial even now as you know, and there may or may not be some errors. Therefore, readers interested in such paper should know this possibility, as all DNA extraction methods contain some errors.

5. It would be good to put a rough estimate of bacterial change in phyla based on ARISA peak change (eg. from Gamma-proteobacteria to alpha-proteobacteria based on length) for NGS substitution.

Unfortunately, we cannot estimate this bacterial change. As you suggested, we are now planning a new experiment to compare the bacterial communities in not only the larvae but also the adult fish. This is because the present study showed us the important period of the formation of intestinal bacteria.

6. As the author mentioned. OTU 429.1 is of my interest. Is there any possible way to know what bacteria it is? It would be powerful to show this bacteria genera/species to consolidate your conclusion in succession of bacterial community.

Unfortunately, we cannot know this bacterial species. However, one of the important results in this manuscript is that bacteria in feeds, controlled by the host fish, do not necessarily establish intestinal bacterial community. We think that the findings obtained in this manuscript are also valuable and appropriate for this journal.

7. Fig S3 (b) : Please indicate color difference (What is yellow and brown group?).

We have addressed it. Thank you. “(b) PCoA constructed using only intestinal samples, the early (yellow group) and late (brown group) stages based on Adonis test (*p* = 0.0001).”

8. Line 266-267 : The sentence "the dominant bacteria are likely to have a great impact on host development, as can be seen from probiotic strategy" is overstated here without evidence based data.

We have toned down this statement as follows: lines 254–255 "The dominant bacteria may have a great impact on host development, as can be seen from probiotic strategy"
